# Optimizing Al_2_O_3_ Ceramic Membrane Heat Exchangers for Enhanced Waste Heat Recovery in MEA-Based CO_2_ Capture

**DOI:** 10.3390/membranes16010043

**Published:** 2026-01-16

**Authors:** Qiufang Cui, Ziyan Ke, Jinman Zhu, Shuai Liu, Shuiping Yan

**Affiliations:** 1 School of Mechatronics and Energy Engineering, NingboTech University, Ningbo 315100, China; 2 College of Engineering, Huazhong Agricultural University, No. 1, Shizishan Street, Hongshan District, Wuhan 430070, China

**Keywords:** CO_2_ capture, waste heat recovery, ceramic membrane heat exchanger, capillary condensation, structural optimization, scale-up behavior

## Abstract

High regeneration energy demand remains a critical barrier to the large-scale deployment of ethanolamine-based (MEA-based) CO_2_ capture. This study adopts an Al_2_O_3_ ceramic-membrane heat exchanger (CMHE) to recover both sensible and latent heat from the stripped gas. Experiments confirm that heat and mass transfer within the CMHE follow a coupled mechanism in which capillary condensation governs trans-membrane water transport, while heat conduction through the ceramic membrane dominates heat transfer, which accounts for more than 80%. Guided by this mechanism, systematic structural optimization was conducted. Alumina was identified as the optimal heat exchanger material due to its combined porosity, thermal conductivity, and corrosion resistance. Among the tested pore sizes, CMHE-4 produces the strongest capillary-condensation enhancement, yielding a heat recovery flux (*q* value) of up to 38.8 MJ/(m^2^ h), which is 4.3% and 304% higher than those of the stainless steel heat exchanger and plastic heat exchanger, respectively. In addition, Length-dependent analyses reveal an inherent trade-off: shorter modules achieved higher *q* (e.g., 14–42% greater for 200-mm vs. 300-mm CMHE-4), whereas longer modules provide greater total recovered heat (*Q*). Scale-up experiments demonstrated pronounced non-linear performance amplification, with a 4 times area increase boosting *q* by only 1.26 times under constant pressure. The techno-economic assessment indicates a simple payback period of ~2.5 months and a significant reduction in net capture cost. Overall, this work establishes key design parameters, validates the governing transport mechanism, and provides a practical, economically grounded framework for implementing high-efficiency CMHEs in MEA-based CO_2_ capture.

## 1. Introduction

Anthropogenic CO_2_ emissions have become one of the most critical drivers of global climate change in the twenty-first century [1]. According to the Sixth Assessment Report of the Intergovernmental Panel on Climate Change (IPCC), the global mean surface temperature has already risen by approximately 1.1 °C above preindustrial levels. To limit warming to within 1.5 °C, global CO_2_ emissions must be reduced by 45% relative to 2010 levels by 2030. Carbon capture, utilization, and storage (CCUS) technologies are, therefore, regarded as essential mitigation pathways, particularly for hard-to-abate sectors such as power generation, steelmaking, and cement production, which together contribute more than 60% of global CO_2_ emissions [2,3,4].

Among the various CCUS technologies, the CO_2_ chemical absorption process using monoethanolamine (MEA) remains the most mature and widely deployed option due to its high CO_2_ loading capacity (0.4–0.5 mol CO_2_ per mol MEA), rapid absorption kinetics, and relatively low capital cost [5,6]. However, the major barrier to its large-scale implementation is the substantial energy required for solvent regeneration [4,6,7]. The decomposition of carbamate species at elevated temperatures (100–120 °C) results in a regeneration energy demand of 3.0–4.5 GJ/t-CO_2_. For a typical coal-fired power plant emitting 10 Mt-CO_2_ annually, the energy required for MEA regeneration alone can reach 3.5 × 10^10^ GJ, equivalent to the annual electricity consumption of one million households—posing significant economic and operational challenges [8].

In a conventional MEA-based capture process, the overhead stream leaving the stripper contains CO_2_ and water vapor at high temperature (above 90 °C) and humidity (the molar of H_2_O(g) is more than 0.5), carrying substantial sensible and latent heat, which normally is removed by a condenser and rejected to cooling water, resulting in heat wasting [9]. Recovering this heat and returning it to the stripper can directly lower the reboiler duty. The “rich-split” modification, where a portion of the CO_2_-rich solvent from the absorber is directed to exchange heat with the stripped gas from the overhead of the stripper, either inside the top of the stripper or in a stainless steel heat exchanger (SSHE), has been proposed as an energy-saving strategy [8,10]. However, the performance of traditional SSHE is limited by low heat-transfer coefficients, susceptibility to corrosion, and large footprint requirements [9,11]. Ceramic membrane heat exchanger (CMHE) has been extensively utilized in desalination and membrane distillation (MD) processes in the past few years because of its superior mechanical strength and outstanding chemical stability. For instance, Rebecca Lee et al. applied hydrophobically modified ceramic membranes to the desalination of high-concentration seawater. Results demonstrated that these membranes maintained excellent performance at operating temperatures as high as 110 °C [12]. Furthermore, Zhang et al. adopted hydrophobic ceramic membranes in membrane distillation. All the studies show the feasibility and robustness of ceramic membranes in corrosive environments [13]. Nowadays, the heat and water recovery from flue gas via CMHE has been widely investigated. Recent advances in porous-membrane-based condensers, particularly CMHEs, have shown promise for intensifying heat and water recovery from flue gas [11,14,15,16,17]. Their nano-pore structure, large specific surface area, and excellent thermal and chemical stability enable capillary condensation of water vapor, substantially enhancing coupled heat–mass transfer [18,19,20].

Despite extensive interest in using CMHEs to recover heat from stripped gas, several critical knowledge gaps remain. Most previous studies optimized one operational or structural variable at a time, overlooking the complex interactions between heat exchanger material, pore size, and membrane length that jointly determine heat and mass transfer performance. For instance, Kim et al. optimized the pore size of ceramic membranes but did not account for the influence of exchanger length on flow resistance or overall energy efficiency [21]. Additionally, the feasibility of linear scale-up, a prerequisite for industrial adoption, has not been adequately assessed. Existing scale-up studies primarily focus on filtration applications, leaving limited insights into heat recovery modules operating with stripped gas. Performance deterioration mechanisms such as flow maldistribution and axial heat loss during scale-up remain insufficiently understood. Furthermore, the industry-scale impacts of material selection, pore size modulation, and tube length on the performance of CMHEs remain poorly understood. Specifically, the synergistic effect between the characteristic length and pore-scale properties, which is crucial for balancing axial driving force decay and transmembrane transport efficiency, has not been adequately addressed.

To address these challenges, this work systematically investigates the effects of membrane pore size and length on CMHE performance under desorbed conditions. Al_2_O_3_ and SiC have been extensively utilized to prepare ceramic membranes as the main components to recover waste heat from flue gas [22]. The SiC ceramic membrane has a better heat recovery performance compared to the Al_2_O_3_ ceramic membrane [22]. However, its widespread industrial application is constrained by demanding fabrication processes and high production costs. Based on that, the Al_2_O_3_ ceramic membrane was adopted in this study. Meanwhile, CMHEs are compared with stainless-steel heat exchangers (SSHEs) and fluoro-plastic heat exchangers (FPHEs) of the same size to highlight material-dependent transport characteristics. Furthermore, the study examines the scale-up behavior of CMHE and elucidates the coupled heat–mass transfer mechanism governing water and energy recovery. The findings establish key design parameters, clarify the constraints associated with non-linear scale-up, and offer practical guidance for integrating CMHEs into the MEA-based CO_2_ chemical absorption process. Ultimately, this work provides a pathway toward reducing the high regeneration energy demand that limits the deployment of MEA-based CO_2_ capture.

## 2. Materials and Methods

### 2.1. Materials

Analytically pure MEA with purity >99.95% provided by Shanghai Lingfeng Chemical Reagent Co., Ltd. (Shanghai, China) was first diluted using ultrapure water to reach the desired MEA concentration (10–40 wt.%). Then aqueous MEA solutions absorbed pure CO_2_ from WISCO Oxygen Co., Ltd. (Shanghai, China) in a typical bubbling reactor to reach the desired CO_2_ loading of about 0.45 mol-CO_2_/mol-MEA, determined by a standard acid-based titration method [15].

**Figure 1:**
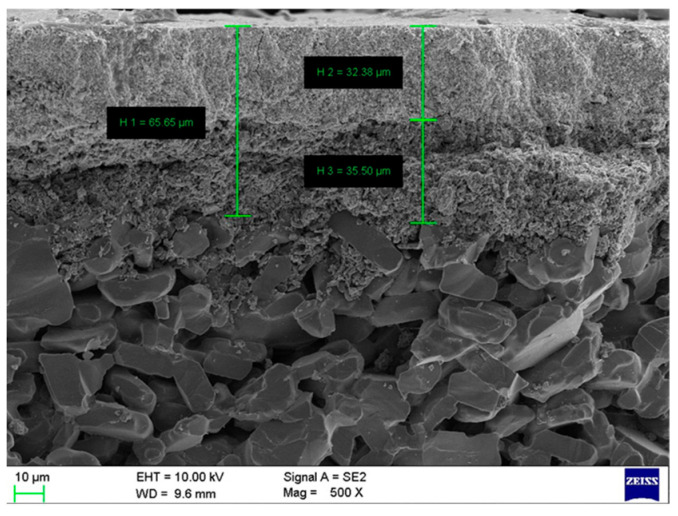
SEM images of membrane tube cross-section (CMHE-10).

**Figure 2:**
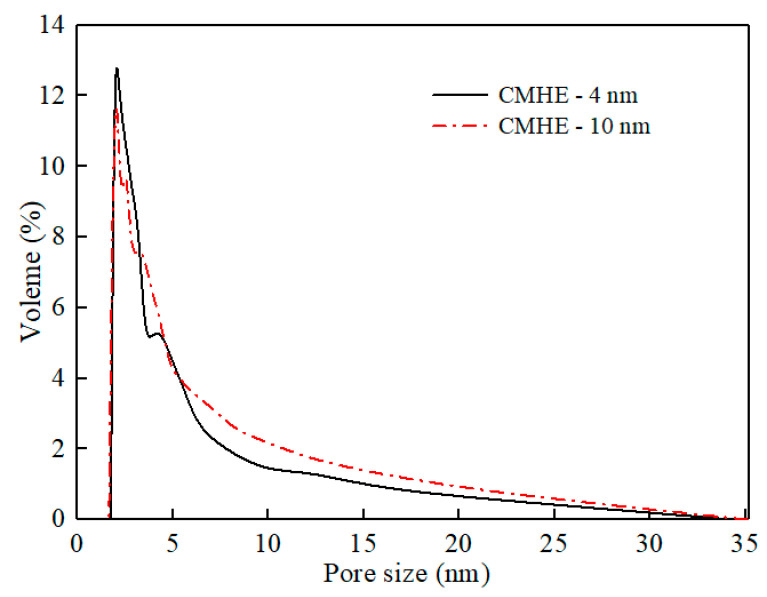
Pore size distribution of the separation layer of 4 nm and 10 nm ceramic membranes.

Ceramic membrane heat exchangers (CMHEs) were assembled from a mono-channel tubular Al_2_O_3_ ceramic membrane with a different separation layer pore size and a stainless steel shell, supported by Nanjing Aiyuqi Membrane Science and Technology Co., Ltd., Nanjing, China. The I.D. and O.D. of the ceramic membrane tubes used in this study are 8 mm and 12 mm, respectively. All ceramic membranes have a tubular structure with a separation layer thickness of 70 μm and a porosity of 23.1% measured with a mercury intrusion porosimeter (AutoPore Micromeritics Instrument Corporation, Norcross, GA, USA). In this study, the membrane pore sizes investigated were 4, 10, 30, and 50 nm. These specific sizes were selected based on the availability of commercial ceramic membranes and their suitability for promoting the capillary condensation of water vapor. Figure 1 and Figure 2 show the SEM images of the membrane tube cross-section and pore size distribution of the membrane. The lengths of the CMHE were 100, 200, 300, and 400 mm. These lengths corresponded to effective membrane areas of 0.0025, 0.005, 0.0075, and 0.01 m^2^, respectively, for the laboratory-scale set-up.

Additionally, to confirm that CMHE has a higher waste heat recovery performance than SSHE, a 304 stainless steel tube with a length of 200 mm and the same inner and outer diameter as the ceramic membrane tube was assembled into the stainless steel shell to construct an SSHE, and the waste heat recovery performance of the SSHE was investigated under the same operating conditions. Furthermore, the waste heat recovery performance of a fluoro-plastic heat exchanger (FPHE) made of polytetrafluoroethylene with the same dimensions as the membrane tube was also examined.

### 2.2. Experimental System

**Figure 3:**
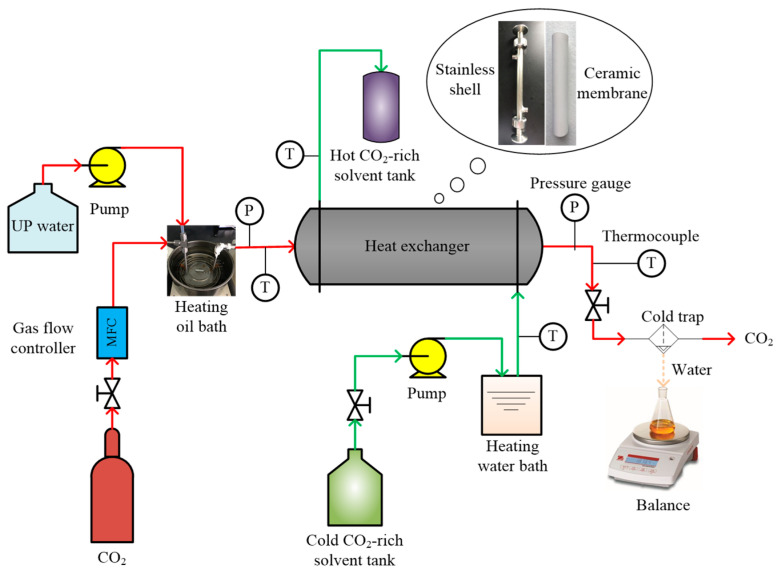
Flow sheet of waste heat recovery from regeneration gas by CMHE in the modified rich-split carbon capture process.

The experimental flow diagram of the heat and water recovery setup from stripped gas using different heat exchangers is shown in Figure 3. The heated CO_2_ from the gas cylinder and water vapor vaporized from ultrapure water in a spiral coil heater immersed in a thermostat oil bath were fully mixed to mimic the hot stripped gas released from the top of the CO_2_ stripper, which is shown in Figure 3. After reaching the desired temperature (~90–110 °C), regulated by precisely controlling the temperature of the thermostat oil bath, the stripped gas was introduced into the lumen of CMHE, placed horizontally. Meanwhile, the cold CO_2_-rich MEA solvent was first heated to 45~65 °C and then passed through the shell of the CMHE counter, currently with the stripped gas. Inside CMHE, the stripped gas will transfer heat and water/vapor to the solvent. After receiving the heat and mass from the stripped gas, the solvent was discharged into a solvent tank. The stripped gas was cooled at the outlet by circulating water at −2 °C in a cold trap to condense the remaining vapor. The condensate in the cold trap was collected and weighed by a precise electronic scale to estimate the total water transfer flux across the ceramic membrane.

### 2.3. Performance Evaluation

Two key performance indicators are employed to assess the heat recovery performance of CMHEs, namely the heat transfer flux and the overall heat transfer coefficient.

Heat recovery flux (*q*) is defined as the heat recovered per unit membrane area per hour. The calculation formula is as follows:

(1) $$q=\frac{Q}{A}$$

where *Q* is the total recovered heat (kJ/h), and A is the effective membrane area (m^2^). The total recovered heat *Q* can be calculated based on the enthalpy change of the MEA solution and the heat related to mass transfer:

(2) $$Q=m\cdot C_{p}\cdot ∆T+J_{w}\cdot A\cdot h_{w}^{T}$$

where *c*_p_ is the constant pressure specific heat capacity of the MEA solution (kJ/(kg K)); m is the mass flow rate of the MEA solution (kg/h); ΔT is the temperature difference between the inlet and outlet of the MEA solution (K). $J_{w}$ is the water transfer flow rate across the membrane (kg/(m^2^ h)); $h_{w}^{T}$ is the specific enthalpy of water at T (K), kJ/kg.

The overall heat transfer coefficient (*U*) characterizes the comprehensive heat transfer efficiency of CMHEs, considering the thermal resistances from the gas side, the membrane, and the solvent side. The calculation formula is based on the log mean temperature difference (LMTD) method:

(3) $$U=\frac{1000\cdot q}{\Delta T_{LMTD}}$$

where $\Delta T_{LMTD}$ is the log mean temperature difference (K), calculated as follows:

(4) $$\Delta T_{m}=\frac{\left(T_{gas}^{in}-T_{sol}^{out})-(T_{gas}^{out}-T_{sol}^{in}\right)}{\ln(\frac{T_{gas}^{in}-T_{sol}^{out}}{T_{gas}^{out}-T_{sol}^{in}})}$$

where $T_{gas}^{in}$ and $T_{gas}^{out}$ are the inlet and outlet temperatures of the hot fluid (rich liquid), respectively (K); $T_{sol}^{in}$ and $T_{sol}^{out}$ are the inlet and outlet temperatures of the cold fluid (lean liquid), respectively (K).

Additionally, *q*_rec_ is also a useful index to analyze the heat recovery performance of CMHE, which can be described using the following equation:

(5) $$q_{rec}=\frac{m_{sol}^{out}\cdot h_{sol}\left(T\right)-m_{sol}^{in}\cdot h_{sol}\left(T\right)}{m_{{CO}_{2}}}$$

where *q*_rec_ is the regeneration energy consumption reduction potential (kJ/kg-CO_2_), $m_{sol}^{out}$ and $m_{sol}^{in}$ are the outlet and inlet solvent mass flow rate (kg/h), $h_{sol}\left(T\right)$ is the specific enthalpy of solvent at T (kJ/kg), $m_{{CO}_{2}}$ is the CO_2_ mass flow rate (kg/h).

## 3. Results and Discussion

### 3.1. Coupled Heat–Mass Transfer Mechanism

#### 3.1.1. Heat and Mass Transfer Mechanism

The heat recovery from stripped gas using a ceramic membrane heat exchanger (CMHE) is governed by a strongly coupled transport process involving vapor condensation, water/vapor migration through the ceramic membrane, and heat conduction across the ceramic membrane [23,24,25]. Previous studies have suggested that this process is dominated by a “capillary condensation” mechanism [26,27]; however, its validity for CO_2_/H_2_O(g) streams in contact with CO_2_-rich MEA solutions has not been conclusively established. In this work, targeted experiments were performed to quantify individual transport pathways and verify the governing mechanism under realistic conditions.

**Figure 4:**
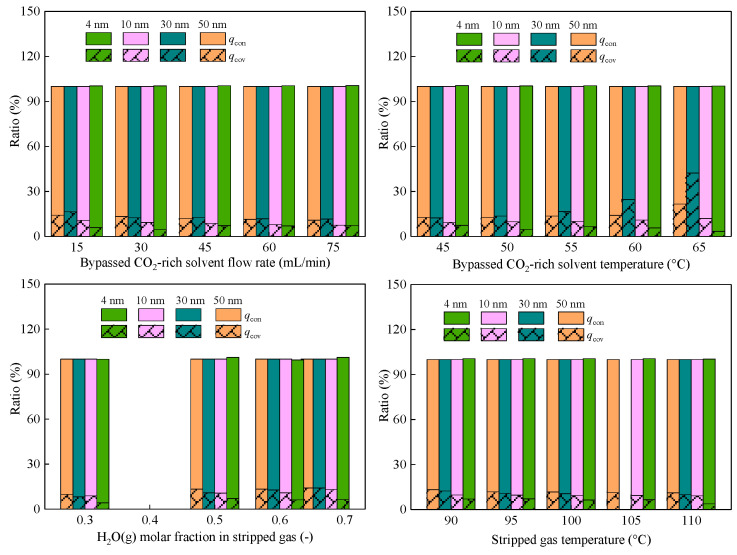
Differences in heat transfer in CMHE with different pore sizes.

Figure 4 presents the decomposed contributions of heat and mass transfer. Liquid water permeation was found to be the predominant mass-transfer route, accounting for 85.09–89.47% of the total water transport in our previous work [23]. Namely, liquid water transfer dominates the mass transfer. Mass transfer will affect heat transfer. Heat transfer data further reveal that membrane conduction (*q*_con_) dominates the heat transfer pathway, consistently contributing more than 80% of the total recovered heat, as can be found in Figure 4. The convective heat (*q*_cov_) associated with water transfer contributes less than 20%, indicating that most latent heat carried by stripped gas is first released before transferring to the solvent side across the membrane. This evidence establishes that membrane conduction affects the heat recovery performance of the ceramic membrane the most. Thus, increasing the membrane thermal conductivity and decreasing the membrane thickness is the most promising way to improve the ceramic membrane performance [16].

#### 3.1.2. Uncertainty and Sensitivity Analysis

To evaluate the reliability of the heat flux decomposition (*q*_con_ vs. *q*_cov_), a systematic uncertainty and sensitivity analysis was conducted. Since *q*_con_ is determined by the temperature gradient and estimated effective thermal conductivity (*λ*_eff_), its accuracy is critical to the mechanistic interpretation.

The uncertainty is estimated using the error propagation law. For the baseline 400 mm membrane case ($T_{gas}^{in}$ = 90.4 °C, $T_{sol}^{in}$ = 44.8 °C), the calculated total heat flux shows a cumulative uncertainty of 4.8%. This indicates that the measurement errors from thermocouples and flow meters do not fundamentally alter the heat transfer trends observed.

Considering the potential variability of *λ*_eff_ in porous ceramic membranes due to varying moisture content and pore structure, a sensitivity analysis was performed by varying the baseline *λ*_eff_ (5.0 W/(m·K)) by ±20%. As summarized in Table 1, although the absolute value of *q*_con_ fluctuates with *λ*_eff_, the contribution of latent heat consistently dominates the process.

The analysis reveals that even under a conservative overestimation of thermal conductivity (+20% *λ*_eff_), the latent heat release from capillary condensation still accounts for approximately 68.7% of the total heat flux. This reinforces the mechanistic conclusion that the heat–mass transfer in the CMHE is primarily governed by phase-change energy release rather than simple conduction.

### 3.2. Material Screening for Heat Exchangers

The physicochemical properties of heat exchanger materials determine their thermal performance, long-term stability, and suitability for operation in corrosive environments. To clarify the advantages of CMHEs in MEA-based regeneration systems, this section compares the performance of three tubular heat exchangers fabricated from alumina ceramic, 304 stainless steel, and unmodified PTFE. All exchangers were tested under the same geometric configurations and identical operating conditions to ensure strict comparability.

#### 3.2.1. Comparative Analysis of Material Performance

**Figure 5:**
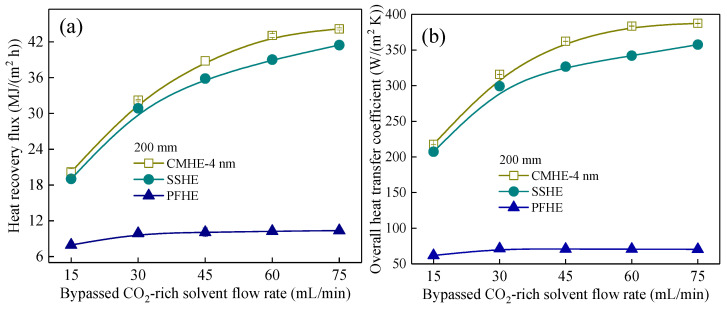
Comparison of the consumption reduction potential of heat exchangers made by different materials under different solvent flow rates. The effect of solvent flow rate on (**a**) heat recovery and (**b**) overall heat transfer coefficient.

Figure 5 presents the variations in waste heat recovery flux (*q*) and overall heat transfer coefficient (U) as a function of rich-solvent split ratio for the three heat exchangers. Although all exchangers exhibit improved performance with increasing liquid flow rate, the CMHE consistently outperforms both the stainless steel heat exchanger (SSHE) and fluoro-plastic heat exchanger (FPHE) across all operating conditions.

Quantitatively, the *q* values of CMHE are 0.28–4.30% higher than those of the SSHE and 140.65–302.81% higher than those of the FPHE. Similarly, its *U* values exceed those of SSHE by 5.02–12.14% and those of FPHE by 251.80–449.45%. These substantial performance differences arise from three synergistic enhancement mechanisms intrinsic to the ceramic membrane:

(1) High thermal conductivity

Heat transfer in CMHE is dominated by membrane conduction (as established in Section 3.1). The ceramic membrane consists mainly of alumina, which exhibits a very high thermal conductivity of ~29.3 W/(m K) compared to 304 stainless steel (~16 W/(m K) and fluoro-plastic (~0.23 W/(m K)), resulting in CMHE having a higher heat transfer coefficient compared to the other two heat exchangers. In this case, more latent and sensible heat can be recovered successfully.

(2) Additional heat transfer related to mass transfer

The porous structure of the ceramic membrane allows the water/vapor transfer into the solvent side. Consequently, the latent or sensible heat carried by the transported water or vapor is inherently transferred to the solvent side. Therefore, the CMHE exhibits superior waste heat recovery performance compared to the other two types of heat exchangers.

(3) Lower gas-phase thermal resistance

The water in the stripped gas side will inevitably condense either on the gas-membrane side or within the membrane pores, resulting in a condensate layer on the stripped gas side, which consequently causes a lower convective heat transfer coefficient. However, condensate can be transferred driven by the trans-membrane pressure difference during the heat and mass transfer process in CMHE, causing a higher overall heat transfer coefficient of CMHE, as shown in Figure 5b.

Together, these advantages explain the superior *q* and *U* values of CMHE, particularly in the environment dominated by vapor condensation.

Additionally, stripped gas is hot, humid, and weakly acidic. Stainless steel is susceptible to corrosion under such conditions, which can reduce its lifetime and alter the heat recovery performance over time [6,11]. However, PTFE is chemically stable, but the extremely low thermal conductivity restricts its applicability [28]. Alumina ceramic offers excellent corrosion resistance, thermal stability, and mechanical strength, ensuring stable long-term operation.

#### 3.2.2. Integrated Performance Evaluation and Implications for Material Selection

The comparative results highlight the distinct strengths and limitations of the three materials. PTFE, despite its excellent corrosion resistance, suffers from inherently poor heat-transfer performance due to its extremely low thermal conductivity and is therefore unsuitable for high-humidity, condensation-driven heat-recovery applications. SSHE remains attractive for recovering heat by conduction. However, the formation of a condensate layer and susceptibility to corrosion significantly degrade its heat recovery performance when handling stripped gas.

In contrast, the CMHE leverages its unique combination of porous architecture, high thermal conductivity, and chemical inertness to achieve a better heat and water recovery performance than SSHE and FPHE. Additionally, CMHE can also recover high-purity condensate from stripped gas, which will flow back to the stripper, resulting in a lower water loss in the CO_2_ stripper [15].

From a material-selection perspective, CMHE could be the superior choice for developing compact, high-efficiency devices to reduce the regeneration energy consumption of MEA-based CO_2_ capture. This conclusion establishes a foundation for the subsequent optimization of membrane pore size and length, which affect the coupled heat–mass transfer performance of CMHE.

### 3.3. Selection of Ceramic Membrane Pore Size

Membrane pore size is a critical parameter that affects capillary condensation and mass transfer across the membrane, driven by the transmembrane pressure difference, which indirectly influences the heat transfer performance of CMHE. CMHE with different pore sizes (4, 10, 30, and 50 nm, labeled by CMHE-4, CMHE-10, CMHE-30, and CMHE-50, respectively) were adopted and investigated in terms of heat-recovery flux (*q*) and water-recovery flux (*J*) to optimize the pore size under different working conditions. Additionally, the engineering implications of CMHEs were also discussed.

#### 3.3.1. Influence of Pore Size on Heat and Water Recovery

**Figure 6:**
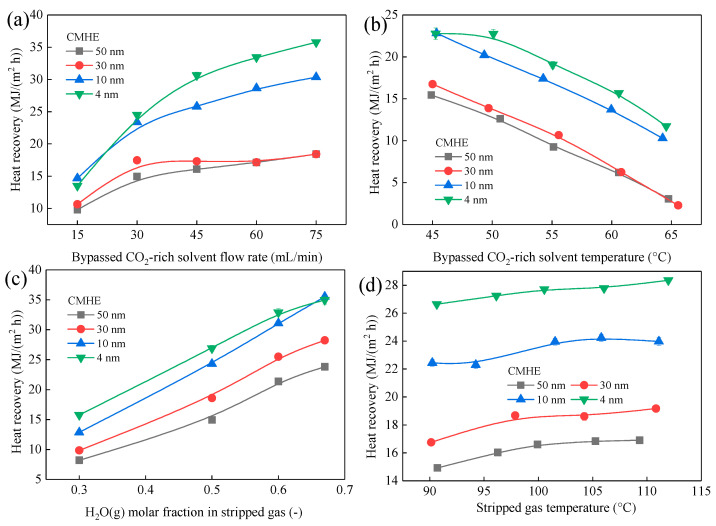
Waste heat recovery performance of CMHEs with different pore sizes under varying rich liquid flow rates. The effect of (**a**) solvent flow rate, (**b**) solvent temperature, (**c**) H_2_O(g) molar fraction, and (**d**) stripped gas temperature on heat recovery.

**Figure 7:**
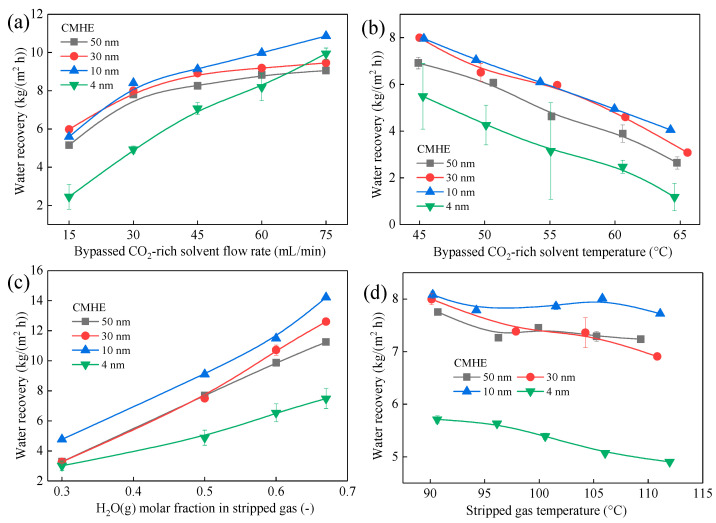
Water recovery performance of CMHEs with different pore sizes under varying rich liquid flow rates. The effect of (**a**) solvent flow rate, (**b**) solvent temperature, (**c**) H_2_O(g) molar fraction, and (**d**) stripped gas temperature on water recovery (The length of CMHEs is 300 mm).

Figure 6 and Figure 7 display the variations in q and J of CMHE with different pore sizes over a range of operating conditions. The heat recovery performance of CMHE increases with pore size, increasing from 4 nm to 50 nm, as can be seen in Figure 6. However, the water recovery performance of CMHE increases first and then decreases with pore size increasing from 4 nm to 50 nm. For example, when the bypassed solvent flow rate is 30 mL/min, CMHE-4 can obtain heat recovery flux of 24.52 MJ/(m^2^ h), which is 1.05 times that of CMHE-10 with a heat recovery flux of 23.37 MJ/(m^2^ h) and 1.64 times that of CMHE-50 with a heat recovery flux of 14.93 MJ/(m^2^ h). However, the water recovery flux of CMHE-4 is 4.92 kg/(m^2^ h), which is 0.63 times that of CMHE-50 with a water recovery flux of 7.80 kg/(m^2^ h) and 0.59 times that of CMHE-10 with a water recovery flux of 8.40 kg/(m^2^ h). The aforementioned results can be explained by capillary condensation in the CMHE with nano-sized pores, which can be found in Section 3.3.2.

#### 3.3.2. Mechanistic Interpretation Based on Capillary-Condensation

According to the Kelvin equation (Equation (5)), smaller pores exhibit significantly reduced saturated vapor pressures *P*_sat_, thereby promoting water condensation at higher temperatures or lower vapor pressures *P*_v_.

(6) $$\ln\left(\frac{P_{v}}{P_{sat}}\right)=\frac{2\gamma V_{m}}{rRT}cos\theta$$

where $P_{v}$ is the vapor pressure over the curved meniscus; $P_{v}$ is saturated vapor pressure; γ is the surface tension of the liquid; $V_{m}$ is Molar volume of the liquid; *r* is pore radius; *R* is ideal gas constant; *T* is absolute temperature; θ is the contact angle.

According to the capillary condensation mechanism in nano-pores, more water tends to condense in smaller-sized membrane pores while the pore size is within 4 nm–50 nm. Additionally, more water condensate means more latent heat can be released. Therefore, more latent heat can be released in the CMHE-4. In this case, more heat can be recovered in CMHE-4 compared to other CMHEs. Consequently, CMHE-4 has the highest heat recovery performance, followed by CMHE-10, CMHE-30, and CMHE-50. Meanwhile, CMHE-10 has the highest water recovery flux, followed by CMHE-10 and CMHE-30, which is a similar trend to that of heat recovery. However, CMHE-4 has a lower water transfer flux due to the higher mass transfer resistance within the smaller membrane pores compared to CMHE-10, even though CMHE-4 can form more condensate. The aforementioned results indicate that the maximum mass flux does not necessarily coincide with the maximum heat flux, as the latter is governed by a more complex interplay between mass transfer and heat transfer pathways.

#### 3.3.3. Conclusions on Pore-Size Selection and Engineering Implications

The aforementioned results clearly show that pore size exerts a dominant influence on the heat recovery performance of CMHEs. For applications aiming to maximize *q* when recovering the waste heat from a vapor-saturated mixture, like stripped gas or flue gas, the CMHE with smaller membrane pores that promote strong capillary condensation is preferred. In this study, CMHE-4 provides the superior heat recovery performance. However, CMHE-4 gets a relatively low water recovery flux compared to CMHE-10. These results highlight that maximizing heat recovery does not correlate with maximizing water flux. Better performance can be achieved when the membrane pore size simultaneously enables (i) strong capillary condensation for more latent heat release and (ii) sufficient permeability to maintain continuous condensate transport. The CMHE-4 aligns with these requirements, delivering the highest q and offering significant advantages in compact, high-efficiency heat-recovery module design. Moreover, the findings regarding pore size provide foundational design principles for scaling CMHEs toward industrial applications, directly guiding membrane fabrication, module configuration, and large-scale system integration.

### 3.4. Selection of Membrane Length

Membrane length is also a key parameter, which fundamentally dictates the residence time of the stripped gas and the driving force of condensation. In this section, the CMHEs with different lengths were adopted to investigate the waste heat recovery performance in terms of heat recovery flux in MJ/(m^2^ h), total recovered heat in MJ/h, and regeneration energy consumption reduction potential in kJ/kg-CO_2_ to meet different engineering requirements. Four different types of length, namely 100, 200, 300, and 400 mm (corresponding to membrane areas of 0.0025–0.01 m^2^), were used in this section.

#### 3.4.1. Distinct Effects of Membrane Length on Area-Based and Total Performance

**Figure 8:**
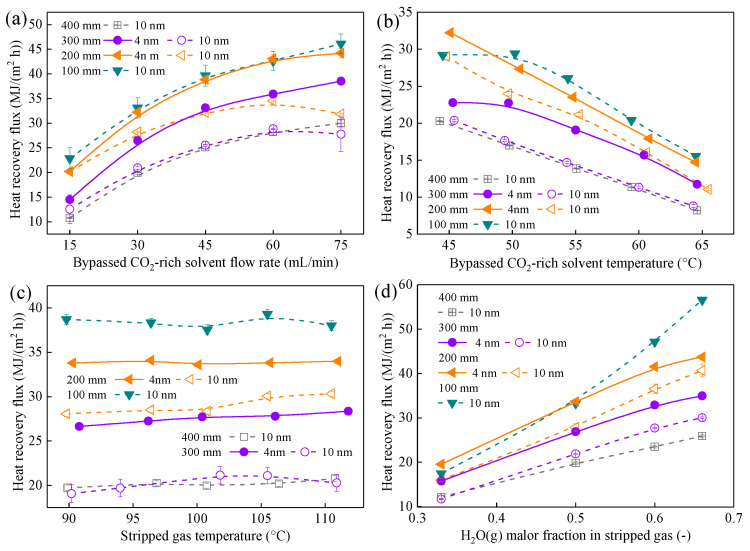
Influence of different operating parameters on the heat transfer flux of CMHE with different specifications. The effect of (**a**) solvent flow rate, (**b**) solvent temperature, (**c**) stripped gas temperature, and (**d**) H_2_O(g) molar fraction on heat recovery flux.

Figure 8 shows that membrane length affects q and Q value differently. Shorter CMHE consistently achieves a higher q value across all operating conditions. For example, the q value of 300-mm CMHE-4 modules is 14.08–41.52% lower than that of the 200-mm modules when the solvent flow rate changes. This reduction arises from the axial decay of the temperature difference and vapor partial-pressure difference as heat and mass transfer proceed along the membrane. Short CMHE maintains higher local driving forces near the inlet, where condensation is most intense, exhibits superior area utilization, and higher q.

To quantitatively demonstrate the axial decay of the driving force, the Log-Mean Temperature Difference (LMTD) for the CMHE with different membrane lengths was calculated based on the inlet and outlet temperatures, which can be seen in Table 2. As the membrane length increases from 100 mm to 400 mm, the LMTD, representing the effective thermal driving force, drops significantly from 31.1 °C to 19.8 °C. This experimental trend confirms that the latter sections of the longer membranes operate under a much smaller temperature gradient, leading to the observed ‘trade-off’ where total recovered heat in MJ/kg increases while the area-normalized heat flux (*q*) decreases.

**Figure 9:**
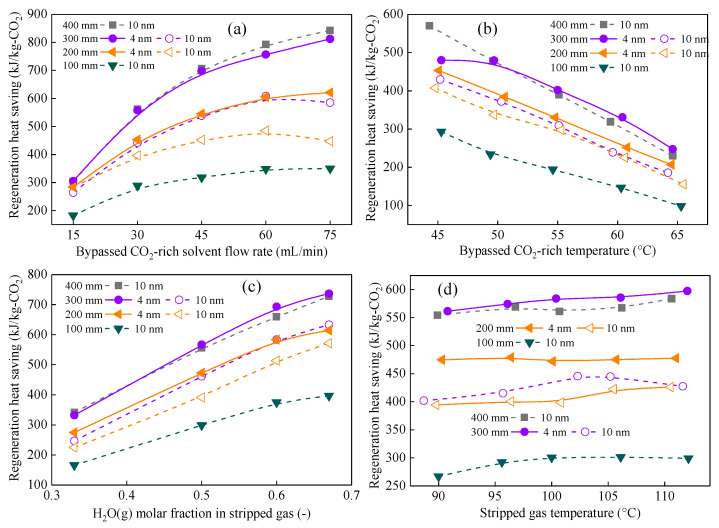
Effect of different operating parameters on the regeneration heat saving potential of CMHE with different specifications. The effect of (**a**) solvent flow rate, (**b**) solvent temperature, (**c**) H_2_O(g) molar fraction, and (**d**) stripped gas temperature on regeneration heat saving.

Additionally, the *q*_rec_ value increases with membrane length (Figure 9). This is because longer CMHEs possess a larger total heat-transfer area, leading to a higher total recovered waste heat (Q). At a constant CO_2_ flow rate, this translates into a higher heat recovery per unit of treated stripped gas. This trend contrasts with that of the q value in MJ/(m^2^ h), which is normalized per unit of membrane area. Clearly, the use of different performance indicators leads to practical discrepancies in evaluating the heat-recovery performance of CMHE. Therefore, the choice of evaluation metrics should be determined based on specific practical requirements in real applications.

#### 3.4.2. Synergistic Effects of Pore Size and Length: Performance-Based Sizing

Combining the pore-size effects with length-dependency highlights a synergistic coupling between micro- and macro-scale design of CMHE. Specifically, the CMHE-4 can obtain the same heat recovery performance as CMHE-10 with a longer length. For instance, a 200-mm CMHE-4 achieves a q value comparable to that of a 300-mm CMHE-10. A 300-mm CMHE-4 delivers total heat recovery similar to that of a 400-mm CMHE-10. These results indicate that optimizing pore size can substantially reduce the membrane length required to recover the waste heat from a mixture with a given space. This characteristic is especially valuable for improving the present industrial systems within a confined footprint.

The heat recovery performance ranking of all CMHEs under all working conditions is as follows: By heat-recovery flux (q): 100-mm CMHE-4 > 200-mm CMHE-4 > 200-mm CMHE-10 > 300-mm CMHE-4 > 300-mm CMHE-10 > 400-mm CMHE-10. By total heat-recovery potential (Q): 300-mm CMHE-4 > 400-mm CMHE-4 > 200-mm CMHE-4 > 300-mm CMHE-10 ≈ 400-mm CMHE-10 > 200-mm CMHE-10 > 100-mm CMHE-10. These rankings indicate that CMHE-4 can be used to achieve superior heat recovery performance compared to other CMHEs regardless of a given space, suggesting that CMHE-4 represents a more economical choice.

#### 3.4.3. Engineering Implications and Conclusions for Length Selection

The selection of membrane length depends on the industrial applications and should be based on a balance between compactness and energy savings. Generally, in cases requiring structural compactness and high volume ratios, CMHE-4 provides a higher heat flux, which is a better option because of the rapid heat transfer performance. For maximizing total heat recovery in industrial applications, longer membranes (300–400 mm) offer superior total heat savings, which are preferable. Additionally, if a higher heat recovery performance and structural compactness of CMHE are required, CMHE-4 might also be the optimal choice. This synergy between pore size and length is essential for developing modular CMHE tailored to varied industrial constraints.

However, in practical industrial cases, it is unfeasible to indefinitely increase the length of the CMHE to achieve higher recovery performance; despite this, CMHE is widely considered to exhibit favorable linear scale-up characteristics. However, the results in the present study (Figure 8 and Figure 9) clearly indicate that a longer CMHE leads to lower utilization efficiency, suggesting the waste heat recovery performance of CMHE may not follow the linear scale-up effect when processing large-scale stripped gas. Consequently, the study in Section 3.5 was conducted to investigate the relationship between membrane area and heat recovery performance.

### 3.5. Scale-Up Behavior

**Figure 10:**
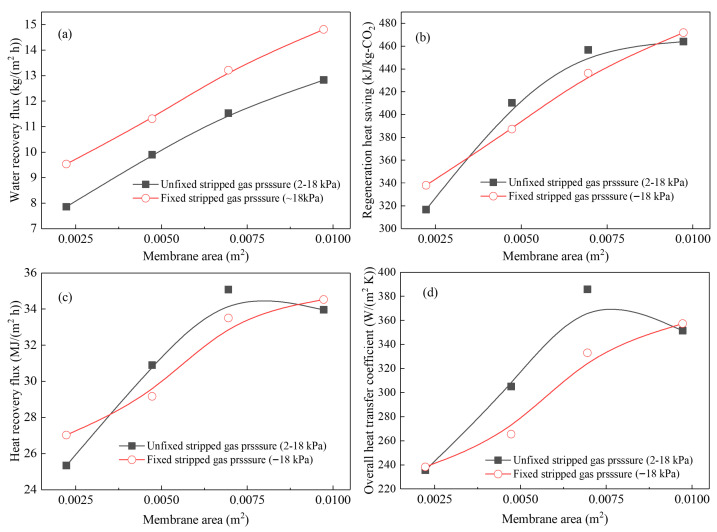
CMHE-4 performance amplification. The varying trends of (**a**) water recovery, (**b**) regeneration heat saving, (**c**) heat recovery flux, and (**d**) overall heat transfer coefficient with membrane area.

A critical question for transitioning CMHE technology from laboratory-scale to industrial implementation is whether the performance of CMHE increases proportionally with the membrane area. To investigate the scalability of the CMHE, the experiments were conducted while maintaining the ratio of the flow rate of stripped gas to solvent. The results can be shown in Figure 10.

#### 3.5.1. Experimental Design and Observation of Non-Linear Scale-Up Behavior

CMHE-4 was used to carry out scale-up experiments. The membrane area was increased from 0.0025 to 0.01 m^2^ by extending the tube length, while maintaining constant superficial gas loading (72 Nm^3^/(m^2^·h)) and stripped gas–solvent flow rate ratio (100:1). Two working conditions were examined: (i) uncontrolled inlet pressure, which changes between 2 and 18 kPa, and (ii) a fixed inlet pressure of approximately 18 kPa. The results in water flux (J), regeneration energy consumption reduction (q_rec_), heat recovery flux (q), and overall heat-transfer coefficient (U) are shown in Figure 10. The results demonstrate a pronounced deviation from linear scale-up. Under constant inlet pressure, increasing membrane area by 4 times resulted in J, Q, q, and U increasing by only 1.56, 1.40, 1.26, and 1.50 times, respectively, substantially below the theoretical 4 times increase expected from the linear scale-up effect. Additionally, when the inlet pressure is not fixed, both q and U values are slightly decreased, by 3.21% and 8.94%, respectively, as the membrane area increased from 0.0075 to 0.01 m^2^, indicating the onset of performance deterioration at larger scales. Notably, the heat recovery performances of CMHE, in terms of q, q_rec_, and U values, are higher when the working pressure is fixed, because of the longer resistance time of stripped gas, higher vapor condensate, and heat convection related to water transfer. For example, when the pressure is fixed, and the membrane area is 0.01 m^2^, the q and q_rec_ values can go up to 34.53 MJ/(m^2^ h) and 471.86 kJ/kg-CO_2_, which are higher than the unfixed pressure scenario. Controlling the stripped gas pressure might be necessary under certain working conditions. These observations reveal that membrane-area enlargement does not translate into proportional gains in heat and mass transfer and that performance may even decline beyond a certain threshold.

#### 3.5.2. Mechanistic Analysis of the Non-Linear Scale-Up Phenomenon

The non-linear behavior arises from several interacting transport phenomena that become increasingly significant as membrane length grows:

(1) Heat and mass transfer driving forces decline with membrane length

As heat and water vapor are transferred along the membrane, both temperature difference and vapor partial pressure difference decrease more rapidly in larger modules. The increased rate of the heat recovery performance of the CMHE decreases with the membrane length, because of the lower temperature difference and driving force difference across the membrane.

(2) Interaction between pressure dynamics and phase-change behavior

In the membrane heat recovery system, pressure fluctuations influence the dew-point temperature and the capillary-condensation behavior of vapor. When membrane length becomes sufficiently large, pressure drop and low vapor concentration jointly alter vapor condensation behavior, leading to changes in the location and intensity of heat–mass transfer zones. These effects are inherently non-linear and cannot be captured by simple geometric scaling.

#### 3.5.3. Key Engineering Implications for Industrial Scale-Up

The findings from the scale-up experiments carry significant implications for industrial CMHE design: large-scale performance cannot be predicted by a single-tube study. The strong non-linearity result indicates that laboratory-scale CMHE performance does not extrapolate linearly to industrial dimensions. Relying on small-scale data risks overestimating system efficiency. The redesigns of the CMHE module- and system-level redesign are essential. Industrial CMHEs must incorporate engineered flow-distribution structures (e.g., optimized headers, multi-pass flow configurations, or parallel module arrays) to ensure uniform utilization of membrane area. Tube bundle geometry and module layout must be reconsidered relative to small-scale designs. Active performance enhancement strategies are required to overcome diminishing returns. Potential strategies include improving membrane-surface transport via hydrophilic treatments, pore-structure tuning, optimizing module geometry to minimize axial ΔT and ΔP decay, incorporating staged condensation, multi-zone heating to redistribute driving forces, or deploying pressure-control systems to stabilize condensation behavior. Identification of scale-up limits guides industrial feasibility. The CMHE performance decline at large membrane areas highlights the existence of a practical scale limit for single-module CMHEs. Industrial systems should therefore favor modular designs, where multiple smaller CMHE units operate in parallel to ensure stable, high-efficiency heat recovery.

Overall, the scale-up study reveals that achieving industrial-scale CMHE performance requires coordinated optimization at the membrane, module, and system levels rather than direct geometric scaling. These insights form a crucial foundation for future engineering development and commercialization efforts.

### 3.6. Comprehensive Performance Comparison with Conventional Heat Exchangers

To fully assess the competitiveness of CMHE, their performance must be compared against established technologies under identical geometric and operational conditions. This section evaluates the optimized 4-nm CMHE against SSHEs and PTFEs in terms of multiple performance criteria, including heat and mass transfer performance, material durability, operational reliability, and compactness.

#### 3.6.1. Comparative Performance Matrix

Table 3 summarizes the multi-dimensional performance comparison among CMHE-4, SSHE, and FPHE. The CMHE exhibits superior performance in all heat- and mass-transfer metrics, particularly in applications involving high humidity and condensation-driven heat recovery. The quantitative values of *q*, *U*, and *J* presented in Table 3 were obtained experimentally under identical operating conditions.

The 4-nm CMHE achieves the highest waste heat recovery flux (*q* = 38.8 MJ/(m^2^ h)), outperforming the SSHE and FPHE by approximately 4.3% and 304%, respectively, when the solvent flow rate is 45 mL/min. Its overall heat-transfer coefficient (*U* = 362 W/(m^2^ K)) also exceeds that of the SSHE (345 W/(m^2^ K)) and the FPHE (66 W/(m^2^ K)). In addition, CMHE can recover high-purity water by 6–12 kg/(m^2^ h), whereas the other cases can not.

The distinctions among the three technologies stem from their respective heat-transfer mechanisms. The CMHE combines membrane conduction with convective heat transfer driven by transmembrane water transfer. In contrast, heat transfer in SSHE and FPHE units occurs only through conduction, which is strongly limited by condensate film accumulation on the gas side. The superior thermal conductivity of alumina and its porous architecture provide the CMHE with a substantial performance advantage.

In terms of durability, alumina ceramics exhibit exceptional resistance to the weakly acidic, high-temperature, high-humidity environment of desorbed gas, whereas stainless steel faces corrosion risks, and PTFE is constrained by its low thermal stability. The ceramic membrane also offers higher volumetric efficiency due to its high specific surface area, allowing more compact module designs.

#### 3.6.2. Analysis of Performance Advantages and Underlying Mechanisms

The superior performance of the CMHE arises from three major enhancement mechanisms, which are related to its material properties and pore structure:

(1) Coupled heat and water recovery through capillary condensation

The porous ceramic membrane surface allows condensed water to permeate through the membrane rather than accumulate as a thick condensate film. This reduces the gas-side thermal resistance encountered in nanopermeation exchangers. Moreover, the transmembrane movement of condensed water introduces an additional convective heat-transfer that carries both latent and sensible heat, a mechanism not present in SSHE or FPHE systems.

(2) Reduced thermal resistance in the gas side due to heat conduction through the ceramic membrane

The majority of heat transfer (>80%) in the CMHE occurs through conduction in the membrane consisting of aluminum with a thermal conductivity of ~29.3 W/(m^2^ K), which is almost double that of stainless steel and vastly higher than plastic. The ceramic membrane can efficiently transfer the latent heat released by vapor from stripped gas, ensuring a high and stable thermal gradient across the membrane.

(3) Material stability enabling long-term, high-temperature, high-humidity operation

Stainless steel is susceptible to corrosion in wet CO_2_ environments due to carbonic acid formation, which may degrade performance over time. PTFE is chemically robust but cannot withstand prolonged exposure to temperatures approaching 110 °C, making it unsuitable for desorbed conditions. Alumina ceramic membrane offers superior thermal and chemical stability, ensuring consistent performance and extended service life.

These three reinforcement effects, namely multiphase transport, high thermal conductivity, and environmental durability, collectively explain why the CMHE achieves significantly higher heat recovery performance while simultaneously enabling water recovery and sustaining long-term operational reliability.

#### 3.6.3. Application Scenarios and Suitability Assessment

The comparative evaluation clarifies the application domains for each heat-exchanger type: CMHEs are ideal for recovering heat and water from vapor-rich or condensable gas streams, such as stripped gas or flue gas. Their porous structure provides exceptional compactness, high volumetric heat-transfer capacity, and water recovery. These characteristics make CMHEs especially suitable for retrofits in space-limited carbon capture processes and for processes requiring high efficiency and long-term stability. Although cost-effective and widely used in industry, SSHEs might be a better choice when dealing with clean, non-condensable gas environments with low corrosion. For high humidity conditions, condensate film and corrosion reduce its competitiveness. Protective coatings or upgraded alloys can address these issues, but at an increased cost and with potential performance penalties. FPHEs excel in strong corrosive environments but are severely limited by poor thermal conductivity and insufficient temperature tolerance. These constraints hinder their application in high temperature mixture like stripped gas.

The CMHE demonstrates superior performance compared to conventional heat exchangers when dealing with stripped gas. Its combined characteristics, including improved heat and mass transfer, material stability, compactness, and the capability to recover water, render it highly suitable for integration into an amine-based CO_2_ capture process.

### 3.7. Techno-Economic Assessment

A simplified techno-economic assessment was performed to examine the feasibility of integrating CMHE-4 into an MEA-based CO_2_ capture process. Based on experimental data, the membrane area required for a representative stripped gas flow rate (0.6 Nm^3^/min) was estimated to be approximately 0.065 m^2^. The corresponding capital and operating expenditures, including membrane procurement, module housing, periodic replacement, additional pumping, etc., were found to be marginal relative to the overall operating cost of the CO_2_ capture unit.

A simplified systematic techno-economic assessment was performed to examine the feasibility of integrating the optimized 4-nm CMHE into an MEA-based CO_2_ capture system. The assessment was based on the following explicit assumptions: ① Membrane cost: 800 USD/m^2^ (based on current quotations for tubular Al_2_O_3_ ceramic membrane with 4-nm pore size). ② Membrane lifespan: 5 years under continuous operation, with annual cleaning required to mitigate fouling from amine aerosols. ③ Integration penalties: Additional pumping power estimated at 0.8 kW per module, and control system costs approximated at 15% of membrane module cost. ④ Steam cost: 25 USD/ton (industrial low-pressure steam). ⑤ Operating time: 8000 h/year.

Based on experimentally measured heat-recovery fluxes, the membrane area required for a representative stripped gas flow rate (0.6 Nm^3^/min) was estimated to be 0.065 m^2^. The corresponding capital expenditure (CAPEX) for the CMHE module, housing, and auxiliary equipment was calculated to be approximately 1200 USD. The annual operational expenditure (OPEX), including pumping energy and cleaning, amounted to about 180 USD/year.

The recovered heat leads to a reduction in reboiler steam demand of ∼0.38 ton/h, corresponding to annual energy savings of ∼7600 USD. Even under conservative assumptions, the payback period is about 2.5 months, and the net present value (NPV) over a 10-year lifetime remains strongly positive.

**Figure 11:**
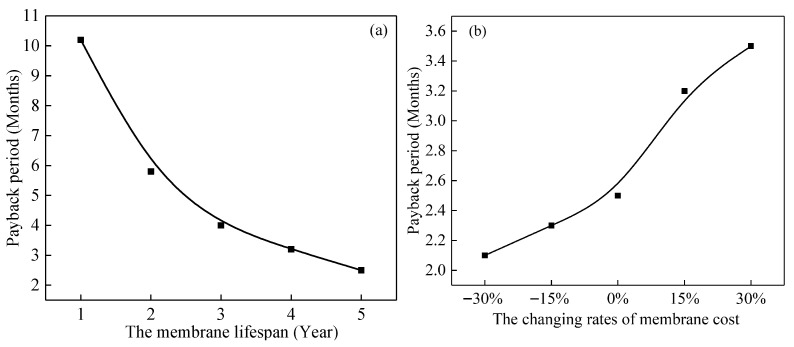
Sensitivity of CMHE payback period to variations in (**a**) lifespan cost and (**b**) membrane.

Sensitivity analysis (Figure 11) was conducted on two key variables: membrane cost and membrane lifespan. If the membrane cost increases by 30%, the payback period extends to about 3.5 months. If the lifespan is reduced to 3 years, the payback period remains under 4 months. In all scenarios, the CMHE integration yields a favorable economic return, with energy saving exceeding incremental costs by more than an order of magnitude.

For a comprehensive comparison, Table 4 summarizes the total cost of ownership (TCO) over 10 years for CMHE, SSHE, and FPHE under identical duty. Although the CMHE has a higher initial CAPEX, its superior energy recovery and lower maintenance (no corrosion-related replacements) result in the lowest TCO, reinforcing its economic attractiveness for long-term deployment.

### 3.8. Industrial Potential and Key Challenges

The current development stage of the CMHE for MEA-based CO_2_ capture process can be assessed as Technology Readiness Level (TRL) 4–5, corresponding to “validation in a laboratory environment” to “validation in a relevant environment.” The present work has demonstrated stable performance under mimicked gas compositions, temperatures, and flow conditions, and has identified key design parameters (material, pore size, length) and scale-up constraints. To advance toward pilot-scale integration (TRL 6–7), the following steps are essential: ① Fabrication and testing of multi-tube CMHE modules (≥10 tubes) to address flow distribution and sealing reliability. ② Long-term (≥1000 h) endurance testing under real flue-gas conditions, including amine aerosols and cyclic operation.③ Development of modular housing designs and standardized ceramic-metal sealing protocols for industrial assembly. ④ Dynamic process-control studies to integrate the CMHE with existing stripper control loops.

The non-linear scale-up behavior observed in Section 3.5 underscores that industrial deployment will require module engineering rather than simple geometric scaling. With continued development targeting the above challenges, the CMHE is positioned as a promising technology for intensifying heat recovery in the carbon capture process. The results show that integrating CMHE into MEA based CO_2_ capture process demonstrates strong potential due to the CMHE’s ability of recovering latent and sensible heat efficiently, maintain stable operation in humid acidic environments, and operate without solvent crossover. Its compact configuration and high volumetric heat-transfer capacity further enhance applicability in retrofitting scenarios where space is limited.

However, several challenges must be addressed for large-scale deployment. First, industrial CMHE modules will require large tube arrays, imposing stringent demands on flow distribution, ceramic-metal sealing, and mechanical robustness under thermal cycling. Second, long-term exposure to amine and degradation products may induce fouling or partial pore blockage, necessitating validated cleaning strategies and fouling control methods. Third, dynamic operating conditions in power-plant environments require coordinated process integration and control to maintain optimal performance. Finally, non-linear scale-up behavior, as revealed in Section 3.5, indicates that industrial designs cannot be achieved through direct geometric scaling, but instead require optimized module design to manage driving-force decay and maldistribution risks.

Overall, while the CMHE exhibits promising technical performance and strong economic potential, addressing these engineering challenges is essential for successful industrial adoption.

## 4. Conclusions

This study systematically investigated the coupled heat–mass transfer mechanism, structural optimization, and scale-up characteristics of Al_2_O_3_ ceramic-membrane heat exchangers (CMHEs) for recovering sensible and latent heat from stripped gas in the MEA-based modification of the CO_2_ chemical absorption process. Experimental decomposition of transport pathways confirms that capillary condensation dominates trans-membrane mass transfer process, while heat conduction through the ceramic membrane dominates the entire heat transfer process in CMHE. Based on capillary condensation, structural optimization results reveal that 4 nm CMHE provides the strongest capillary-driven condensation and gains the highest heat-recovery performance. Membrane length is shown to influence area-based flux and total heat recovery in opposite ways, highlighting the need to balance the compact design of CMHE with requirements. Scale-up results demonstrate distinctly non-linear amplification behavior, indicating that the heat and water recovery performance of CMHE does not increase proportionally with membrane area and that CMHE module design considerations become critical at larger scales. Comparative evaluation further shows that the optimized CMHE outperforms conventional stainless-steel and plastic heat exchangers in both heat and water recovery performances and operational stability. A refined techno-economic assessment, supported by sensitivity analysis and total cost of ownership comparison, confirms that the CMHE offers a payback period of only a few months and significantly reduces the net cost of CO_2_ capture. The technology currently stands at TRL 4–5, with a clear pathway toward pilot-scale validation through module engineering and long-term stability testing. Overall, this work provides not only scientific and design guidelines but also an economically grounded and industrially actionable framework for implementing CMHE-based heat recovery systems in the MEA-based CO_2_ capture process.

## Figures and Tables

**Table 1:** Sensitivity of heat flux decomposition to variations in membrane thermal conductivity (*λ*_eff_) (Data based on 400 mm membrane, baseline case).

Scenario	*λ*_eff_	*q*_con_	Latent Heat Recovered, *q*_lat_ (KW/m^2^)	Contribution (%)
−20% Deviation	4	0.44	1.45	76.70%
Baseline	5	0.55	1.45	72.50%
+20% Deviation	6	0.66	1.45	68.70%

**Table 2:** Driving force attenuation across different membrane lengths (LMTD calculated based on experimental data).

Membrane Length L, (mm)	100	200	300	400
Inlet temperature of stripped gas T_g,in_, (°C)	90.1	90	91	90.4
Outlet temperature of stripped gas T_g,out_, (°C)	83.3	82.6	75.2	79.4
Inlet temperature of MEA solvent T_c,in_, (°C)	44.9	44.2	45.2	44.8
Outlet temperature of MEA solvent T_c,out_, (°C)	63.8	72.5	71.3	77.9
LMTD (°C)	31.1	26.1	24	19.8
Temperature difference between T_g,out_ and T_c,out_, ΔT_exit_, (°C)	38.4	38.4	30	34.6

**Table 3:** Comprehensive Comparison between CMHE and Conventional Heat Exchangers.

Performance Dimension	CMHE-4	SSHE	FPHE	Evaluation and Remarks
Waste-heat recovery flux, *q*, MJ/(m^2^ h)	38.8, High	37.2, Medium	9.6, Low	Under optimal conditions, CMHE achieves ~4.3% higher q than SSHE and ~304% higher than FPHE.
Overall heat-transfer coefficient, U, W/(m^2^ K)	362, High	345, Medium	66, Low	CMHE benefits from a significantly lower total thermal resistance.
Water-recovery capability, J, kg/(m^2^ h)	6–12	None	None	Unique advantage of CMHE: direct condensate recovery with additional latent-heat recovery.
Dominant heat-transfer mechanism	Membrane conduction + convective transport	Pure conduction	Pure conduction	Convective contribution induced by transmembrane mass transfer provides extra enhancement in CMHE.
Corrosion resistance in acidic CO_2_-rich environments	Excellent (chemically inert Al_2_O_3_)	Poor (304 SS prone to corrosion in wet CO_2_)	Excellent (highly corrosion-resistant PTFE)	CMHE and FPHE have substantially longer expected lifetimes in desorbed conditions.
Thermal stability and operating temperature limit	Excellent (>1000 °C)	Good (typically <400 °C)	Moderate (typically <150 °C)	CMHE offers the best thermal robustness for environments with temperature fluctuations.
Fouling resistance	Good (hydrophilic surface)	Moderate	Excellent (hydrophobic PTFE surface)	Hydrophilicity promotes thin water-film formation and reduces adhesion of certain foulants; organic fouling may be more significant on polymeric surfaces.
Volumetric heat-transfer capacity	High (porous structure with a large specific area)	Medium	Low	CMHE can deliver the same heat-recovery duty with a smaller device volume, enabling compact modules.
Material cost	High (precision ceramics)	Low (commodity metals)	Medium (engineering polymers)	Higher initial investment for CMHE is a key barrier to large-scale deployment.
Manufacturing and sealing complexity	Complex (ceramic-metal joining required)	Mature (welding/expansion techniques)	Mature (welding/bonding)	Industrial fabrication and maintenance systems are most well-developed for SSHE and FPHE.

**Table 4:** Total cost of ownership (TCO) comparison over 10 years for three heat-exchanger types (based on a 0.065 m^2^ unit).

Cost Category	CMHE-4	SSHE	FPHE
Capital cost (USD)	1200	900	1000
Annual energy saving (USD)	7600	7280	1880
Annual maintenance (USD)	180	350	200
Replacement cost (10 years)	1200	2700	2000
Total net saving (10 years)	~68,500	~62,300	~8800

Note: Assumes one replacement for SSHE due to corrosion and two for FPHE due to thermal ageing.

## Data Availability

The original contributions presented in the study are included in the article. Further inquiries can be directed to the corresponding author.
